# Design of Oral Sustained-Release Pellets by Modeling and Simulation Approach to Improve Compliance for Repurposing Sobrerol

**DOI:** 10.3390/pharmaceutics14010167

**Published:** 2022-01-11

**Authors:** Chu-Hsun Lu, Yu-Feng Huang, I-Ming Chu

**Affiliations:** 1 Biomedical Technology and Device Research Laboratories, Industrial Technology Research Institute, Hsinchu 30011, Taiwan; markhuang@itri.org.tw; 2 Department of Chemical Engineering, National Tsing Hua University, Hsinchu 30013, Taiwan

**Keywords:** sobrerol, multiple sclerosis, sustained-release, pellets, modeling and simulation, dissolution, pharmacokinetics, pharmacodynamics, IVIVC

## Abstract

Sobrerol, an oral mucolytic agent, in a recent study showed promise for treating multiple sclerosis. A human equivalent dose of 486 mg of sobrerol administered thrice daily (i.e., 1459 mg of daily dose) demonstrated the highest therapeutic efficacy for repurposing use, which also points out the poor compliance of administration. In this study, oral sustained-release pellets of sobrerol were successfully developed with evaluated manufacturing conditions and drug release kinetics. For design of the target drug product, we used a modeling and simulation approach to establish a predictive model of oral pharmacokinetic profile, by exploring the characteristics and correlations corresponding to the pharmacokinetics and pharmacodynamics of sobrerol, such as absorption lag time (0.18 h), time-scaling in vitro–in vivo correlation (*t*_in-vitro_ = 0.494 *t*_in-vivo_ − 0.0904), gastrointestinal transit time (8 h), minimum effective concentration (1.61 μg/mL), and duration of action (12.8 h). Results showed that the frequency of administration and the daily dose remarkably reduced by 33.3% (i.e., from thrice to twice daily) and 22.8%, respectively, which indicates that this prototype approach can be adopted for rapidly developing a modified-release dosage form of sobrerol, with improvement of compliance of administration and therapeutic efficacy.

## 1. Introduction

Sobrerol, developed in the 1970s as a mucolytic agent, is widely used to treat acute or chronic respiratory diseases caused by increased bronchial mucus and obstruction [1]. The recommended dose of sobrerol was 400 mg daily for adults, the longest treatment period was 3 months with a daily dose of 600 mg, and the maximum daily dose was 900 mg for 10 consecutive days. Sobrerol is considered a relatively safe drug with no substantial side effects when administered under guidance [2,3,4]. In addition to respiratory diseases, sobrerol has recently been studied to treat multiple sclerosis, an autoimmune neurological disease [5]. The experimental autoimmune encephalomyelitis (EAE) animal model exhibiting inflammatory demyelination and axonal damage was adopted in the study on multiple sclerosis treatment [6,7], and sobrerol could effectively alleviate EAE symptoms at a dose of 100 mg/kg thrice daily, which is equivalent to the positive control dimethyl fumarate at a dose of 100 mg/kg twice daily. Dimethyl fumarate is the first-line oral drug currently used for treating multiple sclerosis, and its most common adverse events are flushing, diarrhea, abdominal pain, nausea, vomiting, and lymphocyte reduction [8,9]. Compared with dimethyl fumarate, sobrerol has the potential to be safer in treatment. However, the human equivalent dose converted from the animal dose is 486 mg thrice daily (i.e., 1459 mg of daily dose), which considerably exceeds the current therapeutic dose used.

In this study, we developed oral sobrerol sustained-release (SR) pellets to improve compliance for repurposing indications. SR pellets are usually pseudospherical (0.5–1.5 mm in diameter) with a smooth surface, relatively high density, and excellent flowability. SR pellets dosage form is comprised of many small discrete drug delivery units, which can then be filled into capsules or sachets for oral consumption [10,11,12,13]. SR pellets not only have flexible release profiles but can also reduce the risk of dose dumping compared with larger single-unit dosage forms [14,15,16], thus demonstrating the advantages of long-term efficacy and safety.

The Biopharmaceutics Classification System (BCS) is a systematic classification based on the water solubility and intestinal permeability of drugs (as shown in Table S1 of Supplementary Material) [17]. When combined with in vitro dissolution characteristics, this system can be used to determine how fast and how much of a drug is absorbed in the body. To shorten the time and reduce the cost during the development of drug products, in vitro–in vivo correlations (IVIVCs) are often used to express the characteristics of drug products; that is, they accurately predict the correlation of the in vivo pharmacokinetic profiles of drugs with the in vitro dissolution data. If the drugs with a property of high permeability (i.e., BCS class I or II) are stable in the gastrointestinal tract and dissolution is the rate-limiting step during drug absorption, the IVIVCs are expectedly established [18,19]. Therefore, sobrerol can be classified as a BCS class I drug due to high water solubility and intestinal permeability [20,21], which is supported to follow the model of IVIVC, even more in the case of modified-release.

**Scheme 1:**
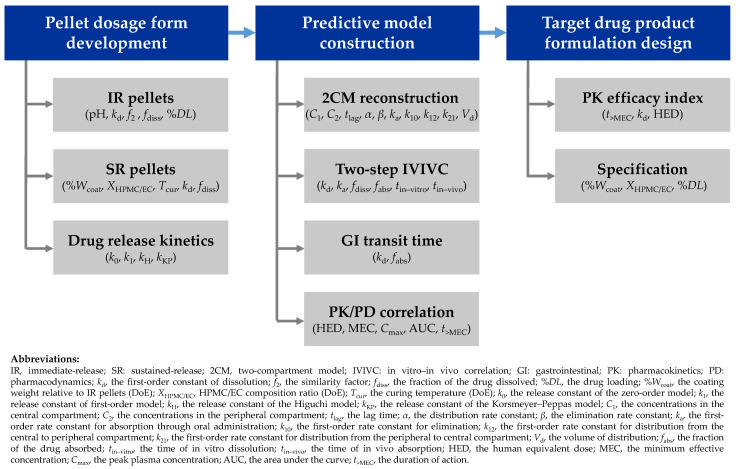
A flowchart for developing oral sobrerol pellet dosage form to improve compliance in repurposing treatment.

As depicted in Scheme 1, we developed pellet dosage forms and evaluated manufacturing conditions and drug release kinetics for various pellets. Subsequently, we predicted the oral pharmacokinetic profiles of pellet dosage forms by adopting modeling and simulation approaches [22,23]. We constructed a predictive model by exploring the characteristics and correlations corresponding to the pharmacokinetics and pharmacodynamics of sobrerol. Finally, we used the constructed predictive model to design the formula and specification of the target drug product of sobrerol to improve compliance of administration on repurposing treatment.

## 2. Materials and Methods

### 2.1. Materials

Sobrerol was purchased from DaeHe Biopharma Co., Ltd. (Gyeonggi-do, Korea). Ethanol and analytical-grade methanol were obtained from Echo Chemical Co., Ltd. (Miaoli, Taiwan). Microcrystalline cellulose (MCC) was obtained from Wei Ming Pharmaceutical Mfg. Co., Ltd. (Taipei, Taiwan). Ethyl cellulose (EC) and hydroxypropyl methyl cellulose (HPMC) were obtained from Ashland Global Specialty Chemicals Inc. (Ashland, KY, USA).

### 2.2. Manufacturing

#### 2.2.1. IR Pellets

Considering that a higher dose is required for repurposing sobrerol, we used the extrusion-spheronization process that can produce high-drug-loaded dosage forms to prepare sobrerol immediate-release (IR) pellets [24,25]. Subsequently, 84 g of sobrerol, 36 g of MCC, and an appropriate amount of water were thoroughly mixed using a mixer (TK-9517, Heas Technology Co., Ltd., New Taipei, Taiwan). The pellets were prepared into a strip shape by using an extruder (SY-BG-120, Shang-Yuh Machine Co., Ltd., New Taipei, Taiwan) at a speed of 40 rpm. Spherical pellets were produced using a spheronizer (SY-SM-40C, Shang-Yuh Machine Co., Ltd., New Taipei, Taiwan) at a speed of 1000 rpm for 1 min. The pellets were finally dried in a hot-air oven (ED-4, Rei Hsiung Enterprise Co., Ltd., New Taipei, Taiwan) at 60 °C for 3 h to obtain IR pellets with 70% drug loading.

#### 2.2.2. SR Pellets

SR pellets were prepared by spraying medical polymers on the IR pellets to form a coating layer. The coating layer was composed of a hydrophobic polymer (EC) and a hydrophilic polymer (HPMC). The formula and preparation conditions were examined using a 3-factor, 3-level design of experiment (3^3^ DoE). A 13-experiment-matrix was generated using Minitab 18 (Minitab LLC., State College, PA, USA) with three factors, namely, coating weight relative to IR pellets (%*W*_coat_, Factor A), HPMC/EC composition ratio (*X*_HPMC/EC_, Factor B), and curing temperature (*T*_cur_, Factor C). Each factor had three levels and was coded from low to high with values of −1, 0, and 1, respectively, and the response indicated the first-order constant of dissolution (*k*_d_ in unit of h^−1^). The 3^3^ DoE matrix of sobrerol SR pellets is presented in Table 1, and actual weights of the components are shown in Table S2 of Supplementary Material.

SR pellets were prepared using the following steps. Various compositions of HPMC and EC were dissolved in 50% (*v/v*) of ethanol to prepare a spraying solution. Subsequently, 180 g of IR pellets (diameter from 500 to 710 μm) were coated with the spraying solution using a fluidized bed granulator (FGA-16, Ohkawara Kakohki Co., Ltd., Kanagawa-ken, Japan) at a feed rate of 0.9–1.4 g/min; other operating conditions were as follows: air pressure, 1.0–1.6 kg/cm^2^; inlet air temperature, 28–33 °C; and exhaust air temperature, 20–23 °C. Finally, the coated pellets were cured in an oven (DO45, Dogger Scientific Co., Ltd., New Taipei, Taiwan) at a set temperature for 6 h. Finally, 13 types of sobrerol SR pellets were obtained.

### 2.3. Characterization

#### 2.3.1. Scanning Electron Microscopy (SEM)

The morphological properties of IR and SR pellets were investigated using an SEM (SU8010, Hitachi Ltd., Tokyo, Japan). The cross-sectional view of the pellet was obtained by cutting the SR pellet with a blade. SEM images were acquired at an accelerated voltage of 10 kV under different magnifications.

#### 2.3.2. High-Performance Liquid Chromatography (HPLC)

Sobrerol was assayed through HPLC (Waters Alliance 2695, Waters Corporation, Milford, MA, USA) with C18 column (Inertsil ODS-2, GL Sciences Inc., Tokyo, Japan). Samples were diluted with 50% (*v/v*) of methanol from 100 μL to 1 mL and filtered through 0.45-μm filtration membranes. The operating conditions were as follows: detection wavelength of UV, 210 nm; flow rate, 0.8 mL/min; injection volume, 10 μL; and mobile phase, 50% (*v/v*) of methanol [26]. Verification of a calibration curve (the R^2^ is not <0.995) and reproducibility (the relative standard deviation of peak areas is not >2.5% in 5 sets of injection) is conducted for system suitability before samples assayed.

#### 2.3.3. Dissolution Test

The dissolution test was performed using USP apparatus 1 (the basket method). We poured 1000 mL of dissolution medium (i.e., pure water; pH 1.2, 4.5, or 6.8 of buffer solutions) into the vessel of the dissolution tester (DT-6, Shin-Kwang Precision Industry, New Taipei, Taiwan). Subsequently, we dropped IR pellets containing 100–130 mg of sobrerol into the basket placed in the dissolution medium, with stirring at 75 rpm. The whole process was performed at 37 ± 0.5 °C. Sampling was performed at 5, 10, 15, 30, 45, 60, 75, 90, and 120 min. For SR pellets, each type was sampled for evaluation at 0.5, 1, 2, 4, 6, and 8 h, and the dissolution medium used was pure water. Except for the aforementioned parameters, other operating conditions were the same as those used for IR pellets.

#### 2.3.4. The Drug Release Mechanism

The fraction of the drug dissolved (*f*_diss_) was calculated for each pellet dosage form. The calculation of the similarity factor (*f*_2_) for IR pellets is shown in Section S2 of Supplementary Material [27]. The drug release mechanism for SR pellets was fitted using four kinetic models (zero-order, first-order, Higuchi, and Korsmeyer–Peppas) as shown in Table S3 of Supplementary Material [28,29].

### 2.4. Establishing the Predictive Model

#### 2.4.1. Reconstruction of the Sobrerol Pharmacokinetic Model

According to a previous study, the plasma concentrations of a single oral dose of 300 mg of sobrerol at 0.25, 0.5, 1, 2, 3, 4, 6, and 8 h were 1.01, 2.82, 3.77, 2.02, 1.41, 1.05, 0.63, and 0.37 μg/mL, respectively [20]. Similarly, using the oral two-compartment model derived from mass balance methods (as depicted in Scheme S1 of Supplementary Material) and considering the lag time of drug absorption, pharmacokinetic parameters were reconstructed using WinNonlin 4.1 (Certara Inc., Princeton, NJ, USA). The oral two-compartment model is described by the following equations [30]:

(1) $$\begin{array}{c} C_{1}\left(t\right)=\frac{k_{a}FD}{V_{d}}(\frac{\left(k_{21}-\alpha \right)}{\left(\beta -\alpha \right)\left(k_{a}-\alpha \right)}e^{-\alpha \left(t-t_{\mathrm{lag}}\right)}+\frac{\left(k_{21}-\beta \right)}{\left(k_{a}-\beta \right)\left(\alpha -\beta \right)}e^{-\beta \left(t-t_{\mathrm{lag}}\right)}+\\ \frac{\left(k_{21}-k_{a}\right)}{\left(\alpha -k_{a}\right)\left(\beta -k_{a}\right)}e^{-k_{a}\left(t-t_{\mathrm{lag}}\right)}) \end{array}$$

(2) $$\begin{array}{cc} C_{2}\left(t\right)=\frac{k_{a}k_{12}FD}{V_{d}} & (\frac{1}{\left(\beta -\alpha \right)\left(k_{a}-\alpha \right)}e^{-\alpha \left(t-t_{\mathrm{lag}}\right)}\\ & +\frac{1}{\left(k_{a}-\beta \right)\left(\alpha -\beta \right)}e^{-\beta \left(t-t_{\mathrm{lag}}\right)}\\ & +\frac{1}{\left(\alpha -k_{a}\right)\left(\beta -k_{a}\right)}e^{-k_{a}\left(t-t_{\mathrm{lag}}\right)}) \end{array}$$

where *C*_1_ and *C*_2_ (μg/mL) are the concentrations in the central and peripheral compartments, respectively, at time *t*, *k*_a_ (h^−1^) is the first-order rate constant for absorption through oral administration, *F* is bioavailability, *D* (μg) is the dose, *V*_d_ (mL) is the volume of distribution, *k*_12_ (h^−1^) and *k*_21_ (h^−1^) are the first-order rate constants for distribution from the central to peripheral compartment and back, respectively, *k*_10_ (h^−1^) is the first-order rate constant for elimination, *α* (h^−1^) is the distribution rate constant, *β* (h^−1^) is the elimination rate constant, *t* (h) is time, and *t*_lag_ (h) is the lag time.

#### 2.4.2. Exploring the IVIVC of Sobrerol

The two-step approach was used to construct a valid IVIVC of sobrerol [31,32]. In the first step, the pharmacokinetic profile shown in Section 2.4.1 was transformed to the in vivo fraction of the drug absorbed (*f*_abs_) by deconvolution, and a time-scaling IVIVC was explored by relating the times of in vitro dissolution (*t*_in-vitro_) and those of in vivo absorption (*t*_in-vivo_) at equivalent fractions. In the second step, the first-step *f*_abs_ was reconverted by convolution to generate a pharmacokinetic profile that was comparable to the original one for model validation. Equations (3) and (4) were used for the deconvolution and calculation of *f*_abs_, respectively, and Equation (5) was used for convolution to predict in vivo pharmacokinetic profiles [33], which are described as follows:

(3) $$∆D\left(t\right)=\left\{∆C_{1}\left(t\right)+\left(k_{10}C_{1}\left(t\right)+k_{12}C_{1}\left(t\right)-k_{21}C_{2}\left(t\right)\right)\times ∆t\;\right\}\times V_{d}$$

(4) $$f_{\mathrm{abs}}\left(t\right)=\frac{\sum ∆D\left(t\right)}{D}$$

(5) $$C_{1}\left(t\right)=\overset{t}{\underset{i=0}{\sum }}\left\{\frac{D\times ∆f_{\mathrm{abs}}\left(i\right)}{V_{d}}\left(\frac{\left(k_{21}-\alpha \right)}{\left(\beta -\alpha \right)}e^{-\alpha \left(t-i\right)}+\frac{\left(k_{21}-\beta \right)}{\left(\alpha -\beta \right)}e^{-\beta \left(t-i\right)}\right)\right\}$$

where ∆*D* (μg) is the drug absorbed at time *t*, ∆*C*_1_ (μg/mL) is the concentration change in the central compartment at time *t*, *f*_abs_ and ∆*f*_abs_ are the accumulated and partial fraction absorbed, respectively, at time *t*, and *i* (h) is the absorption onset time of the partial fraction absorbed.

#### 2.4.3. Consideration of the Gastrointestinal Transit Time

The absorption process of oral drugs in the gastrointestinal tract is considerably complicated, and the small intestine is the major site of drug absorption. Therefore, the gastrointestinal transit time should be considered during the development of SR dosage forms to ensure the extent of the release and absorption of the drug. The gastric transit can range from 0 to 2 h in the fasting state, and the transit time in the small intestine can range from 2 to 6 h [34]. The gastrointestinal transit time is estimated to be 8 h for sobrerol pharmacokinetic profile simulation. The plasma concentration during absorption in 8 h follows Equation (1), and that after 8 h is ruled by the intravenous two-compartment pharmacokinetic model, which can be determined using Equation (6):

(6) $$C_{1}\left(t\right)=\frac{D}{V_{d}}\left(\frac{\left(k_{21}-\alpha \right)}{\left(\beta -\alpha \right)}e^{-\alpha t}+\frac{\left(k_{21}-\beta \right)}{\left(\alpha -\beta \right)}e^{-\beta t}\right)$$

#### 2.4.4. Finding the Pharmacokinetic Efficacy Index

The human equivalent dose (HED) and expected therapeutic efficacy (inhibition of symptoms) of sobrerol for multiple sclerosis treatment were derived from EAE animal tests performed in a previous study (C57BL/6 mice with EAE induced by MOG peptides were administered sobrerol for 14 days after onset. ** *p* < 0.01 and *** *p* < 0.001 compared with the results for vehicle determined using Student’s *t*-test) [5]. On the last day after onset (i.e., the plateau phase), low to high doses were used in this study for vehicle (thrice daily), namely, 25 mg/kg (thrice daily), 30 mg/kg (once daily), 100 mg/kg (thrice daily), 150 mg/kg (twice daily), and 300 mg/kg (once daily), and the corresponding EAE scores were 4.00 ± 0.22, 3.70 ± 0.15, 3.38 ± 0.27, 2.36 ± 0.27 **, 2.55 ± 0.27 **, and 2.78 ± 0.25 ***, respectively. The HED conversion based on the body surface area is shown below [35]:

(7) $$\mathrm{HED}\left(\mathrm{mg}\right)=\mathrm{Animal}\;\mathrm{dose}\left(\frac{\mathrm{mg}}{\mathrm{kg}}\right)\times \frac{\mathrm{Animal}\;K_{m}}{\mathrm{Human}\;K_{m}}\times \mathrm{human}\;\mathrm{body}\;\mathrm{weight}\;\left(\mathrm{kg}\right)$$

where animal *K*_m_ (mouse) and human *K*_m_ (adults) are 3 and 37, respectively, and the human body weight is considered 60 kg for adults. Subsequently, the HED was used to simulate pharmacokinetic profiles by using Equation (1), and the peak plasma concentration (*C*_max_) of the highest HED with no significant difference was assumed to be the minimum effective concentration (MEC). Three pharmacokinetic efficacy indexes, namely, the *C*_max_, the area under the curve (AUC), and the duration of action (*t*_>MEC_) of each pharmacokinetic profile, were calculated to determine the pharmacokinetics/pharmacodynamics correlation [36]. In this study, Excel 2016 (Microsoft Corporation, Redmond, WA, USA) was used for calculating data and drawing figures.

## 3. Results and Discussion

### 3.1. Preparation of IR/SR Pellets and Dissolution Study

**Figure 1:**
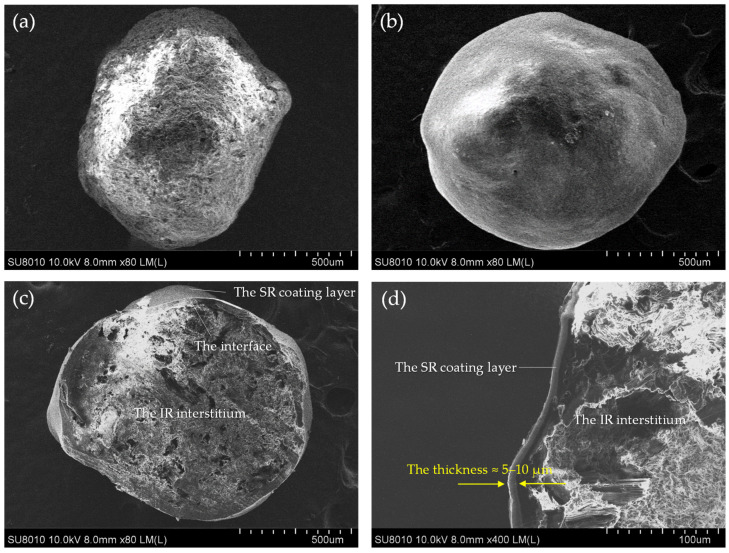
Morphological properties of IR and SR pellets determined using SEM: (**a**) the appearance of IR pellets; (**b**) the appearance of SR pellets; (**c**) the cross-sectional view of SR pellets; and (**d**) the estimation of the thickness of the SR coating layer to be 5–10 μm.

Sobrerol IR pellets were formed in a matrix structure, and the drug loading was more than 70% by total weight. Sobrerol was dispersed in the interstitium of MCC, with a rough and fiber-scattering appearance (Figure 1a). SR pellets were produced from IR pellets with HPMC/EC coated on the surface, which formed a smooth and continuous coating layer with SR function (Figure 1b). In the cross-sectional view, the interstitium of SR pellets showed an irregular crystal arrangement and tiny pores, and a clear interface between the IR and SR coating layer could be observed (Figure 1c). The thickness of the SR coating layer was approximately 5–10 μm (Figure 1d).

As shown in Figure S1 of Supplementary Material, the *f*_diss_ of IR pellets in media with different pH values all exceeded 80% at 30 min and reached the plateau value of 95–100% at 60 min. No degradation was observed, indicating that sobrerol was stable even under pH changes in the gastrointestinal tract and is thus suitable for establishing an IVIVC. The similarity *f*_2_ of the dissolution curves of IR pellets was more than 50 (Table S4 of Supplementary Material), indicating a high similarity of dissolution between various pH media. Thus, water was chosen as the dissolution medium in the following study, and the first-order *k*_d_ of IR pellets was calculated as 4.99 h^−1^.

**Figure 2:**
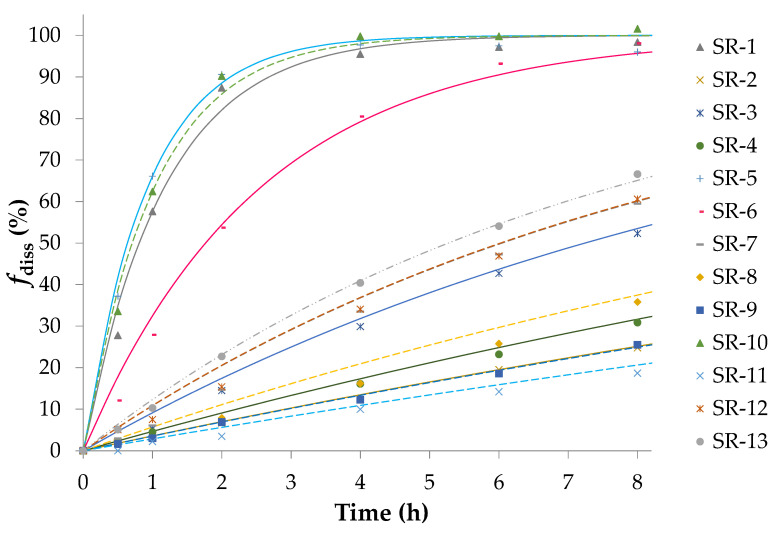
Dissolution tests of sobrerol SR pellets and the first-order fitting curves. The first-order model could most favorably explain the drug release of SR pellets in the 0–8-h interval.

Figure 2 shows the result of the dissolution test for 13 types of SR pellets. Analysis of DoE indicated that Factor B (*X*_HPMC/EC_) exerted a critical effect on SR pellets (i.e., the higher the *X*_HPMC/EC_ is, the higher is the dissolution rate), and a strong interaction was noted between Factor A (%*W*_coat_) and Factor B. Moreover, the 13 fitting curves were well plotted. Among the kinetic models, the first-order model could most favorably explain the drug release of SR pellets in the 0–8-h interval. The first-order *k*_d_ values calculated from high to low were 1.082 h^−1^ (SR-5), 0.978 h^−1^ (SR-10), 0.858 h^−1^ (SR-1), 0.393 h^−1^ (SR-6), 0.131 h^−1^ (SR-13), 0.115 h^−1^ (SR-12), 0.115 h^−1^ (SR-7), 0.096 h^−1^ (SR-3), 0.059 h^−1^ (SR-8), 0.048 h^−1^ (SR-4), 0.036 h^−1^ (SR-2), 0.036 h^−1^ (SR-9), and 0.029 h^−1^ (SR-11), respectively. Additional analytical results of the DoE are provided in Figure S2 of Supplementary Material, and an example for designing a drug product with the target *k*_d_ is discussed in Section 3.3. Dissolution mechanisms are provided in Table S5 and Figure S3 of Supplementary Material.

### 3.2. Establishment of the Predictive Model

#### 3.2.1. Reconstruction of the Sobrerol Pharmacokinetic Model with Lag Time

A sobrerol two-compartment pharmacokinetic model associated with the lag time of drug absorption was established, with *V*_d_ of 44.94 L/h, *k*_12_ of 0.82 h^−1^, *k*_21_ of 0.95 h^−1^, *k*_a_ of 2.40 h^−1^, *α* of 2.08 h^−1^, *β* of 0.26 h^−1^, and *t*_lag_ of 0.18 h. The aforementioned kinetic parameters were incorporated in Equations (1) and (2), and the simulated plasma concentrations over time are shown in Figure S4 of Supplementary Material. The fitting coefficients (R^2^) calculated from the reconstructed model and a previous study [20] were 0.9796 and 0.9257, respectively, indicating that the sobrerol pharmacokinetic model with lag time had more explanatory power.

#### 3.2.2. Time-Scaling IVIVC of Sobrerol

**Figure 3:**
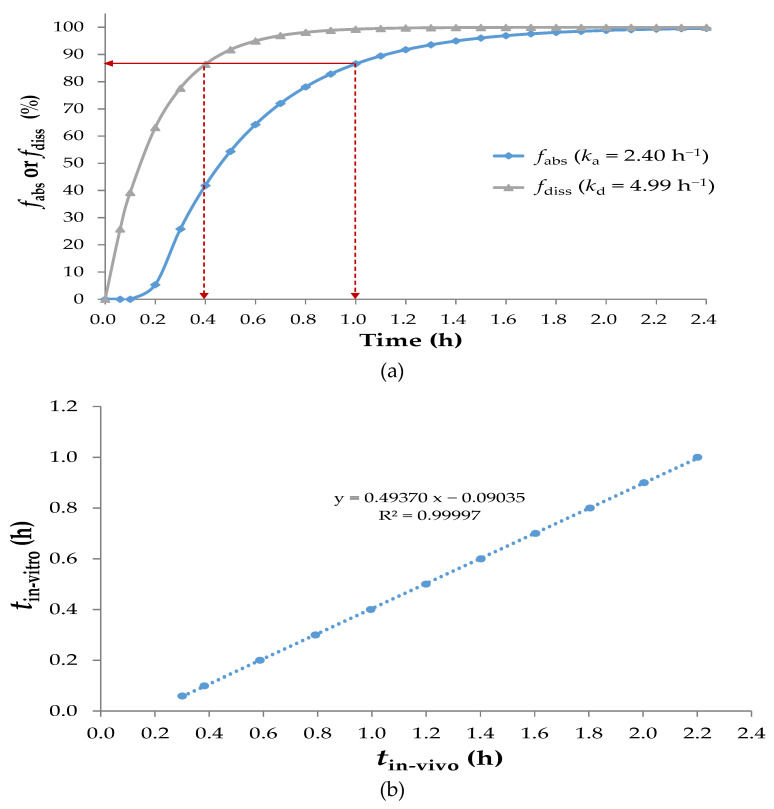
(**a**) The in vivo *f*_abs_ and in vitro *f*_diss_ versus time; (**b**) A linear regression of *t*_in-vitro_ for dissolution versus *t*_in-vivo_ for absorption. Indicating that sobrerol had a favorable time-scaling IVIVC.

The in vivo fraction of the drug absorbed (*f*_abs_), which was obtained by deconvolving the reconstructed sobrerol pharmacokinetic profile, is shown in Figure 3a. Sobrerol orally administered had an *f*_abs_ of >85% in 1 h; thus, it could be classified as a fast-absorbing drug. A satisfactory linear regression of dissolution *t*_in-vitro_ versus absorption *t*_in-vivo_ is shown in Figure 3b, indicating that sobrerol had a favorable time-scaling IVIVC (*t*_in-vitro_ = 0.494 *t*_in-vivo_ − 0.0904). Based on this correlation, a corresponding *f*_abs_ at a certain time could be predicted through in vitro dissolution (e.g., *f*_diss_ at 0.4 h is equivalent to *f*_abs_ at 1.0 h). In the second step, as shown in Figure S5 of Supplementary Material, a reconverted pharmacokinetic profile obtained by convolution fitted well to the original one, showing validation of the IVIVC model. A relationship between *k*_d_ and *k*_a_ (*k*_a_ = 0.478 *k*_d_ + 0.0229) was also verified as shown in Figure S6 of Supplementary Material; *k*_a_ in Equation (1) could be substituted by *k*_d_, making its application easier. In this study, a concept of time-scaling IVIVC involving the IR dosage form of sobrerol was proved, and assuming that any modified-release one mimics the model as well.

#### 3.2.3. Pharmacokinetic Profiles Derived with Gastrointestinal Transit Time

**Figure 4:**
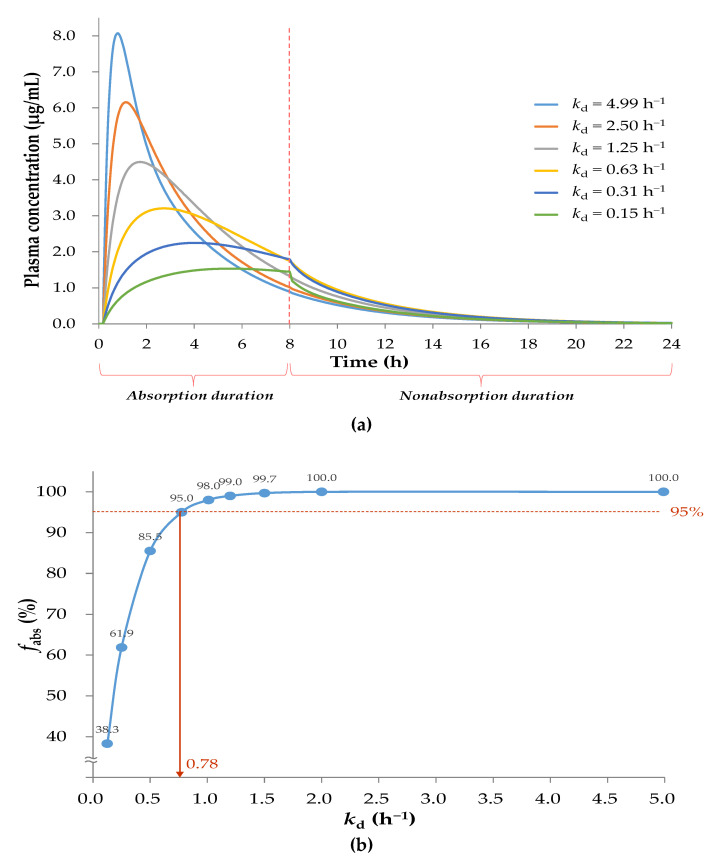
(**a**) Simulation of sobrerol oral pharmacokinetic profiles with various *k*_d_ values in consideration of gastrointestinal transit time of 8 h; (**b**) The correlation of *f*_abs_ at 24 h with corresponding *k*_d_. In case of the *f*_abs_ is >95% by 24 h, the *k*_d_ should not be <0.78 h^−1^.

Considering the factor of gastrointestinal transit time, the extent of drug absorption is limited. As shown in Figure 4a, the pharmacokinetic profile during the 0–8 h of absorption followed the oral two-compartment pharmacokinetic model, and that of the absorbed drug during the duration of nonabsorption followed the intravenous two-compartment pharmacokinetic model. As shown in Figure 4b, *f*_abs_ values decreased with a decrease in *k*_d_ values. For example, in the case of the target *f*_abs_ is >95% by 24 h, the *k*_d_ should not be <0.78 h^−1^.

#### 3.2.4. Pharmacokinetic Efficacy Index of Sobrerol

**Figure 5:**
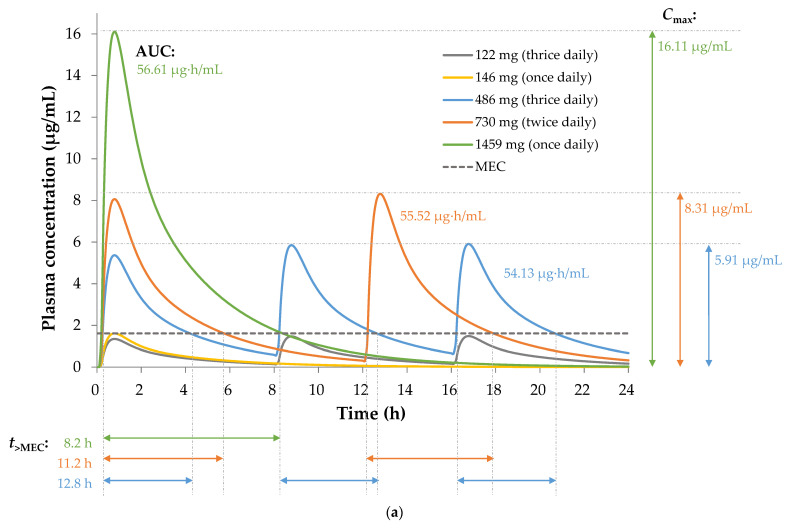

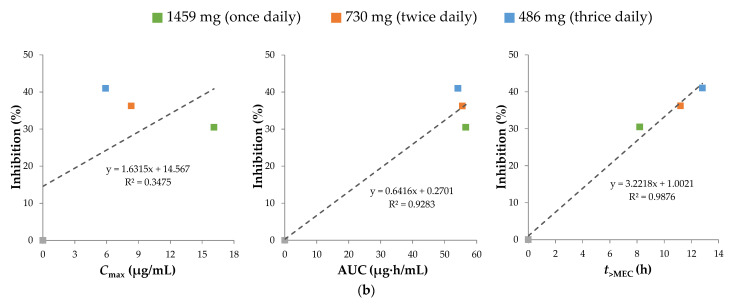
(**a**) Simulation for oral pharmacokinetic profiles of sobrerol with the minimum effective concentration (MEC) of 1.61 μg/mL, and calculation for pharmacokinetic efficacy indexes (*C*_max_, AUC, and *t*_>MEC_) of administration routes with total daily dose of 1459 mg during 24 h. (**b**) Linear regression of each pharmacokinetic efficacy index versus the inhibition rate, showing that time dependence is responsible for the pharmacokinetics/pharmacodynamics correlation of sobrerol.

For repurposing sobrerol, the human equivalent doses (HED) and expected symptom inhibition in humans are shown in Table 2, and the related simulated oral pharmacokinetic profiles of administration routes are shown in Figure 5a. No significant therapeutic efficacy was observed for an HED of 122 mg (thrice daily) or 146 mg (once daily), and we considered the peak concentration of an HED of 146 mg as the minimum effective concentration (MEC), which is 1.61 μg/mL. Symptom inhibitions from high to low were observed with doses of 486 mg (thrice daily), 730 mg (twice daily), and 1459 mg (once daily), which have the same daily dose of 1459 mg. The pharmacokinetic efficacy indexes (i.e., *C*_max_, AUC, and *t*_>MEC_) calculated are also shown in Table 2, and the pharmacokinetics/pharmacodynamics correlation determined by the linear regression of each index versus the inhibition rate is shown in Figure 5b. Among them, *t*_>MEC_ exhibited the highest correlation with therapeutic efficacy, indicating that this is most likely a time-dependent mechanism of treatment, which explains the observation of higher efficacy at a higher frequency of administration under the same daily dose of the drug. Therefore, we used *t*_>MEC_ as the pharmacokinetic efficacy index in the predictive model.

### 3.3. Design of SR Pellets

**Figure 6:**
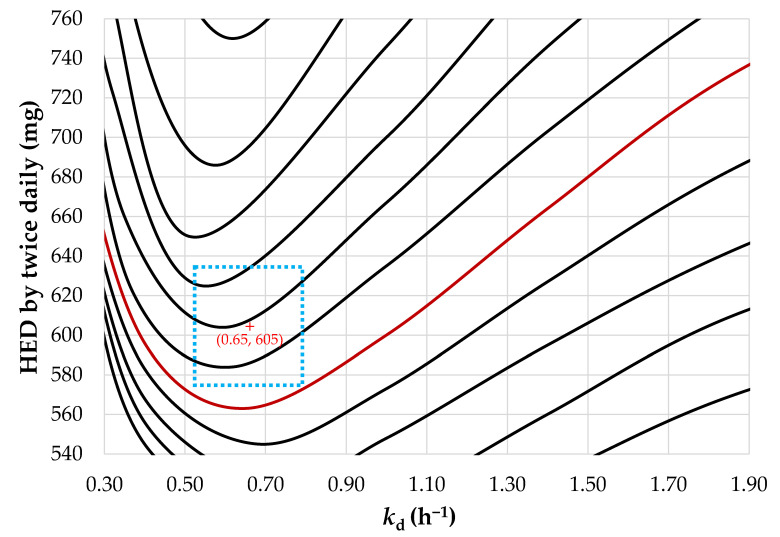
A contour plot of the pharmacokinetic efficacy index (*t*_>MEC_) corresponding to constants of dissolution (*k*_d_) and human equivalent dose (HED). The red curve represents the *t*_>MEC_ of 12.8 h, which the SR dosage form requires to exceed for a consistent or higher efficacy of the drug product. The blue dotted-line area is as an example as the acceptable specification of the target drug product.

In Section 3.2, the characteristics of and the correlation between the pharmacokinetics and pharmacodynamics of sobrerol are discussed, including the pharmacokinetic profile with the lag time, the correlation between the dissolution and absorption of the drug, the gastrointestinal transit time, and the pharmacokinetic efficacy index. Subsequently, the predictive model of oral pharmacokinetic profile for repurposing sobrerol was established. Figure 6 presents the contour plot of the pharmacokinetic efficacy index *t*_>MEC_ generated using the predictive model, where the x-axis is *k*_d_, the y-axis is the HED by twice daily, and *t*_>MEC_ corresponds to the variables of *k*_d_ and HED. The original administration route (i.e., 486 mg by thrice daily) exhibits the highest efficacy, with a *t*_>MEC_ of 12.8 h (shown as a red curve), and the SR dosage form is required to exceed the 12.8-h curve to ensure a consistent or higher efficacy of the drug product. The minimum HED is 563 mg (twice daily) at *k*_d_ of 0.65 h^−1^ when compared with the original administration route; this not only reduces the frequency of administration from thrice to twice a day (by 33.3%) but also the daily dose from 1459 to 1126 mg (by 22.8%), thus considerably improving compliance of administration.

**Figure 7:**
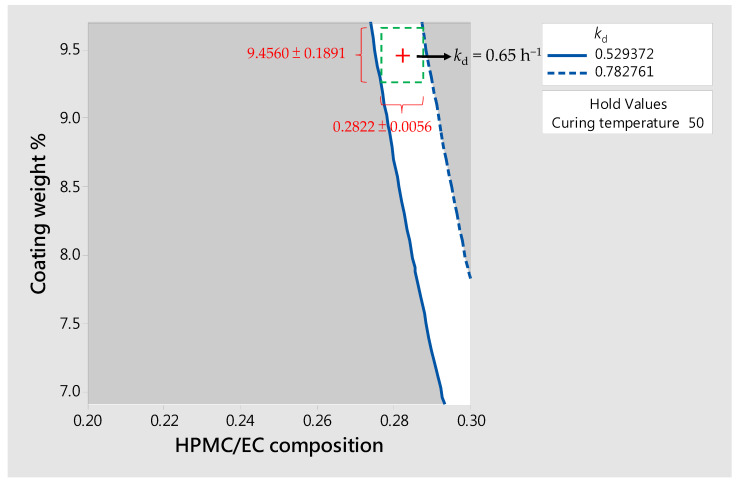
A contour plot of constants of dissolution (*k*_d_) predicted by the DoE. The boundary conditions to the target *k*_d_ of SR pellets is shown by the green dotted-line area, that is, the acceptable specification of *k*_d_ is 0.65 ± 0.13 h^−1^.

However, the tolerance of drug production must be considered in practice. Deviations may result from various factors, such as coating uniformity or drug loading among batches; these factors can affect the dissolution rate and daily dose of the drug. Assuming that the allowable deviation for coating conditions (i.e., *X*_HPMC/EC_ and %*W*_coat_) is 2% and that for drug loading is 5%, the boundary conditions to the target *k*_d_ (i.e., 0.65 h^−1^) of SR pellets predicted using the DoE are 0.2822 ± 0.0056 for *X*_HPMC/EC_ and 9.4560 ± 0.1891% for %*W*_coat_, as shown by the green dotted-line area in Figure 7. In other words, the acceptable specifications of the drug product are tending towards 0.65 ± 0.13 h^−1^ for *k*_d_ and 605 ± 30 mg for HED by twice daily, as shown by the blue dotted-line area in Figure 6.

**Figure 8:**
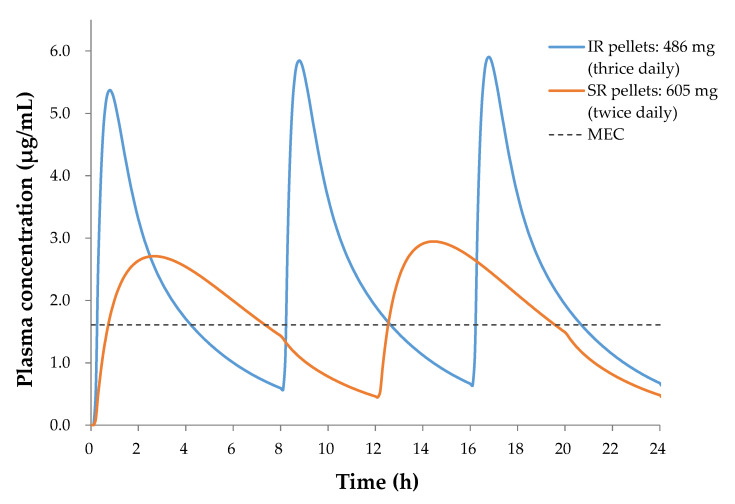
Comparison of pharmacokinetic profiles for the IR pellets (4.99 h^−1^ of *k*_d_) and the SR pellets (0.65 h^−1^ of *k*_d_), indicating that the SR pellets is advantageous from the perspectives of both compliance and efficacy improvement.

Finally, we compared the simulated oral pharmacokinetic profiles of IR pellets with a dose of 486 mg thrice daily and target SR pellets with a dose of 605 mg twice daily, and determined that *t*_>MEC_ values were 12.8 and 13.6 h, respectively, as shown in Figure 8. The *t*_>MEC_ of SR pellets is longer than that of IR ones, indicating that the SR dosage form is advantageous from the perspectives of both compliance and efficacy improvement.

## 4. Conclusions

In this study, we successfully developed high-drug-loaded IR and SR pellets of sobrerol. The proposed manufacturing process is simple with high commercial feasibility. *X*_HPMC/EC_ was determined as the main factor for SR pellet release, and the release followed a first-order model. In addition, a predictive model of oral pharmacokinetic profile based on a time-scaling IVIVC and a time-dependent mechanism for repurposing sobrerol was first established. The predictive model estimated that SR pellets could achieve the goal of improving compliance of administration and therapeutic efficacy in terms of the frequency of administration and dose, that is, taking the drug twice daily and reducing the dose by up to 22.8%. The study also demonstrated a prototype approach of modeling and simulation for rapid design of a drug product, which can reduce the requirement of animal experiments and accelerate research into the clinical status. Future work could further match-up with various pellets of release characteristics to establish an oral multiparticulate system for example, which is highly expandable in medicated application.

## Figures and Tables

**Table 1:** The 3^3^ DoE matrix of sobrerol SR pellets for a total of 13 experiments.

Code	Factor A ^1^ %*W*_coat_	Factor B ^2^ *X*_HPMC/EC_	Factor C ^3^ *T*_cur_
SR-1	0	+1	+1
SR-2	+1	−1	0
SR-3	+1	0	+1
SR-4	−1	−1	0
SR-5	0	+1	−1
SR-6	−1	+1	0
SR-7	0	0	0
SR-8	+1	0	−1
SR-9	0	−1	−1
SR-10	+1	+1	0
SR-11	0	−1	+1
SR-12	−1	0	+1
SR-13	−1	0	−1

^1^ Values of level of −1, 0, 1 corresponding to Factor A: 6.9%, 8.3%, and 9.7 %. ^2^ Values of level of −1, 0, 1 corresponding to Factor B: 0.20, 0.25, and 0.30. ^3^ Values of level of −1, 0, 1 corresponding to Factor C: 50 °C, 60 °C, and 70 °C.

**Table 2:** Exploring for pharmacokinetics/pharmacodynamics correlation of sobrerol: Human plasma simulation and calculation of pharmacokinetic efficacy indexes including *C*_max_, AUC, and *t*_>MEC_.

EAE Animal Tests				Human Plasma Simulations				
In Vivo Dose (mg/kg)	Frequency (Daily)	EAE Score ^1,2^		HED ^3^ (mg)	Inhibition ^4,5^ (%)	*C*_max_ (μg/mL)	AUC (μg·h/mL)	*t*_>MEC_ (h)
Vehicle	thrice	4.00 ± 0.22		-	-	-	-	-
25	thrice	3.70 ± 0.15		122	N/S	1.48	13.59	0.0
30	once	3.38 ± 0.27		146	N/S	1.61	5.67	0.0
100	thrice	2.36 ± 0.27 **		486	41.00	5.91	54.13	12.8
150	twice	2.55 ± 0.27 **		730	36.25	8.31	55.52	11.2
300	once	2.78 ± 0.25 ***		1459	30.50	16.11	56.61	8.2

^1^ EAE score: EAE signs on the last day after onset (the plateau phase). ^2^ ** *p* < 0.01 and *** *p* < 0.001 compared with the vehicle determined using Student’s *t*-test. ^3^ HED: Human equivalent dose conversed from the EAE animal dose, with a human body weight of 60 kg. ^4^ Inhibition: Decreased ratio of the EAE score compared with the vehicle. ^5^ N/S: No significant difference compared with the vehicle.

## Data Availability

Not applicable.

## Supplementary Materials

The following are available online at https://www.mdpi.com/article/10.3390/pharmaceutics14010167/s1, Table S1: BCS classification, Table S2: The formula of SR pellets, Table S3: Kinetic models for drug release study, Table S4: The similarity of the dissolution curves of IR pellets, Table S5: Results for drug release kinetics of SR pellets, Scheme S1: Oral two-compartment model and IVIVC, Figure S1: Results for dissolution of IR pellets, Figure S2: Results for DoE of SR pellets, Figure S3: Fitting plots for SR pellets with various kinetic models, Figure S4: Reconstruction of the sobrerol pharmacokinetic model with lag time, Figure S5: Validation of the time-scaling IVIVC model, Figure S6: The calibration curve of rate constants of in vitro dissolution and in vivo absorption.
