# Supplementary material for: Design of Oral Sustained-Release Pellets by Modeling and Simulation Approach to Improve Compliance for Repurposing Sobrerol
Source: Pharmaceutics. 2022 Jan 11;14(1):167. doi: 10.3390/pharmaceutics14010167 (PMC8777650; doi:10.3390/pharmaceutics14010167)
Supplement: Supplementary file 1 [file pharmaceutics-14-00167-s001.zip › pharmaceutics-1522538-supplementary.pdf]

pharmaceutics

Supplementary Material:

Design of Oral Sustained-release Pellets by Modeling and Simulation Approach to Improve Compliance for Repurposing Sobrerol

Chu-Hsun Lu, Yu-Feng Huang and I-Ming Chu

1. Introduction

Table S1. BCS classification and expected IVIVC for drug products, and sobrerol can be classified as a BCS class I drug.

Class	Solubility	Permeability	IVIVC expectation
**I**	High	High	Possible (if dissolution is rate limiting)
**II**	Low	High	Expected
**III**	High	Low	Little or no IVIVC
**IV**	Low	Low	Little or no IVIVC

a. Definition by the FDA: A drug substance is considered highly soluble when the highest strength is soluble in 250 mL or less of aqueous media within the pH range of 1–6.8 at 37℃±1℃. A drug substance is considered to be highly permeable when the systemic BA or the extent of absorption in humans is determined to be 85% or more of an administered dose based on a mass balance determination (along with evidence showing the stability of the drug in the gastrointestinal tract) or in comparison with an intravenous reference dose.

b. The daily dose of sobrerol used for repurposing (1459 mg) could be fully dissolved in 250 mL of water below the saturated solubility of 32 mg/mL; this met the definition of high solubility in the BCS classification. In addition, a previous study indicated that sobrerol has a high bioavailability of >85% through the oral administration route compared with an intravenous reference dose. These findings indicate that sobrerol can be classified as a BCS class I drug, and an IVIVC can be established.

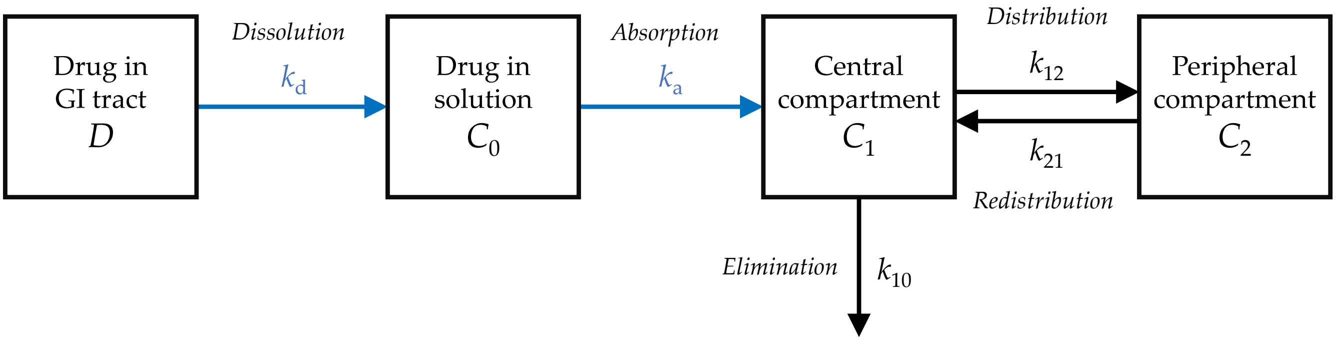

Scheme S1. Oral two-compartment model guided by rate constants of dissolution, absorption, distribution, redistribution, and elimination of the drug. If the BCS class I drugs are stable in the gastrointestinal tract and the drug release is the rate-limiting step during drug absorption, the IVIVCs are expected, e.g., establishing the correlation between the rate constants of in vitro dissolution and in vivo absorption.

2. Materials and methods

Table S2. The actual weights of the coating composition based on 180 g of IR pellets and process conditions for the preparation of SR pellets by using the 3^3 DoE matrix.

Code	Total weight of Coating layer (g)	HPMC in coating layer (g)	EC in coating layer (g)	Curing temperature (℃)
SR-1	15.0	3.46	11.54	70
SR-2	17.5	2.92	14.58	60
SR-3	17.5	3.50	14.00	70
SR-4	12.5	2.08	10.42	60
SR-5	15.0	3.46	11.54	50
SR-6	12.5	2.88	9.62	60
SR-7	15.0	3.00	12.00	60
SR-8	17.5	3.50	14.00	50
SR-9	15.0	2.50	12.50	50
SR-10	17.5	4.04	13.46	60
SR-11	15.0	2.50	12.50	70
SR-12	12.5	2.50	10.00	70
SR-13	12.5	2.50	10.00	50

Similarity factor (f_2) is generally used to examine the equivalency of the dissolution profiles of prepared formulations. In this study, the dissolution test was performed to compare the in vitro dissolution profiles of IR pellets in various dissolution media. The similarity factor (f_2) was calculated using the following equation:

$$f_2 = 50 \log\left\{\left(1 + \frac{1}{n}\sum_{t=1}^{n}(R_t - T_t)^2\right)^{-0.5} \times 100\right\}$$

where n is the number of data points, R_t and T_t are the percentages of drug dissolved in reference and other tests, respectively, at time t. For different dissolution profiles, similarity factors should range from 50 to 100.

Table S3. Kinetic models used to assess drug release data.

Kinetic models	Kinetic equations	Plot equations
Zero-order	$X_t = k_0 t$	$X_t = k_0 t$
First-order	$X_t = 1 - e^{-k_1 t}$	$-ln(1 - X_t) = k_1 t$
Higuchi	$X_t = k_H t^{0.5}$	$X_t = k_H t^{0.5}$
Korsmeyer–Peppas	$X_t = k_{KP} t^n$	$ln(X_t) = n \cdot ln(t) + ln(k_{KP})$

X_t: amount ratio of drug released in time t; k_0: the release constant in the zero-order model; k_1: the release constant in first-order model; k_H: the release constant in the Higuchi model; k_{KP}: the release constant in the Korsmeyer–Peppas model.

3. Evaluation for IR pellets

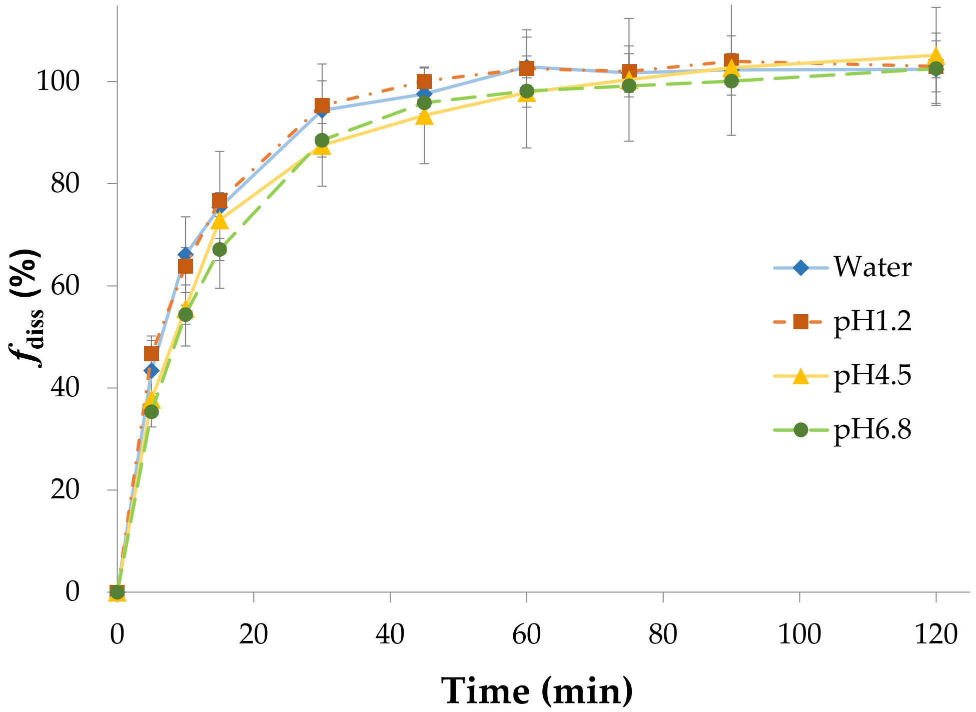

Figure S1. Dissolution tests of sobrerol IR pellets in various dissolution media. No degradation was observed, indicating that sobrerol was stable even under pH changes in the gastrointestinal tract.

Table S4. The similarity f_2 of the dissolution curves of IR pellets in various pH media.

**Reference**	Water	pH1.2	pH4.5	pH6.8	Average
**Water**	-	82.5	60.9	57.6	67.0
**pH1.2**	82.5	-	59.0	55.5	65.7
**pH4.5**	60.9	59.0	-	74.9	65.0
**pH6.8**	57.6	55.5	74.9	-	62.7

Similarity f_2 of the dissolution curves of IR pellets was >50, indicating a high similarity of dissolution in various media with different pH values, and the highest f_2 was observed when water was used as the reference standard in calculations. Therefore, water was used as the dissolution medium in the following dissolution tests of SR pellets.

4. Results of DoE

SR pellets designed using 3^3 DoE can be classified into three groups: the fast-dissolution group with >90% fractions dissolved (i.e., SR-1, SR-5, SR-6, and SR-10), the middle-dissolution group with between 50% and 90% fractions dissolved (i.e., SR-3, SR-7, SR-12, and SR-13), and the slow-dissolution group with <50% fractions dissolved (i.e. SR-2, SR-4, SR-8, SR-9, and SR-11). The effects and interaction of experimental factors were examined using Minitab 18, and the formulation and preparation conditions were further optimized. The analysis results showed that Factor B (HPMC/EC composition ratio) was a critical factor, whereas factor A (coating weight % relative to IR pellets) and factor C (curing temperature) were weak factors (Figure S2a). The effect of Factor C was almost negligible (thus the curing process could be operated at a fixed and lower temperature of 50°C), whereas Factors A and B exhibited a stronger interaction (Figure S2b). The contour plot of the response (k_d) with variables of Factors A and B (keeping Factor C constant) is shown in Figure S2c, and the regression equation is as below:

$k_d = 10.58 - 0.490 \text{ Factor A} - 75.7 \text{ Factor B} - 0.00263 \text{ Factor C} + 131.7 \text{ Factor B} \times \text{Factor B} + 2.13 \text{ Factor A} \times \text{Factor B}$

which can be used to predict k_d when designing target SR pellets.

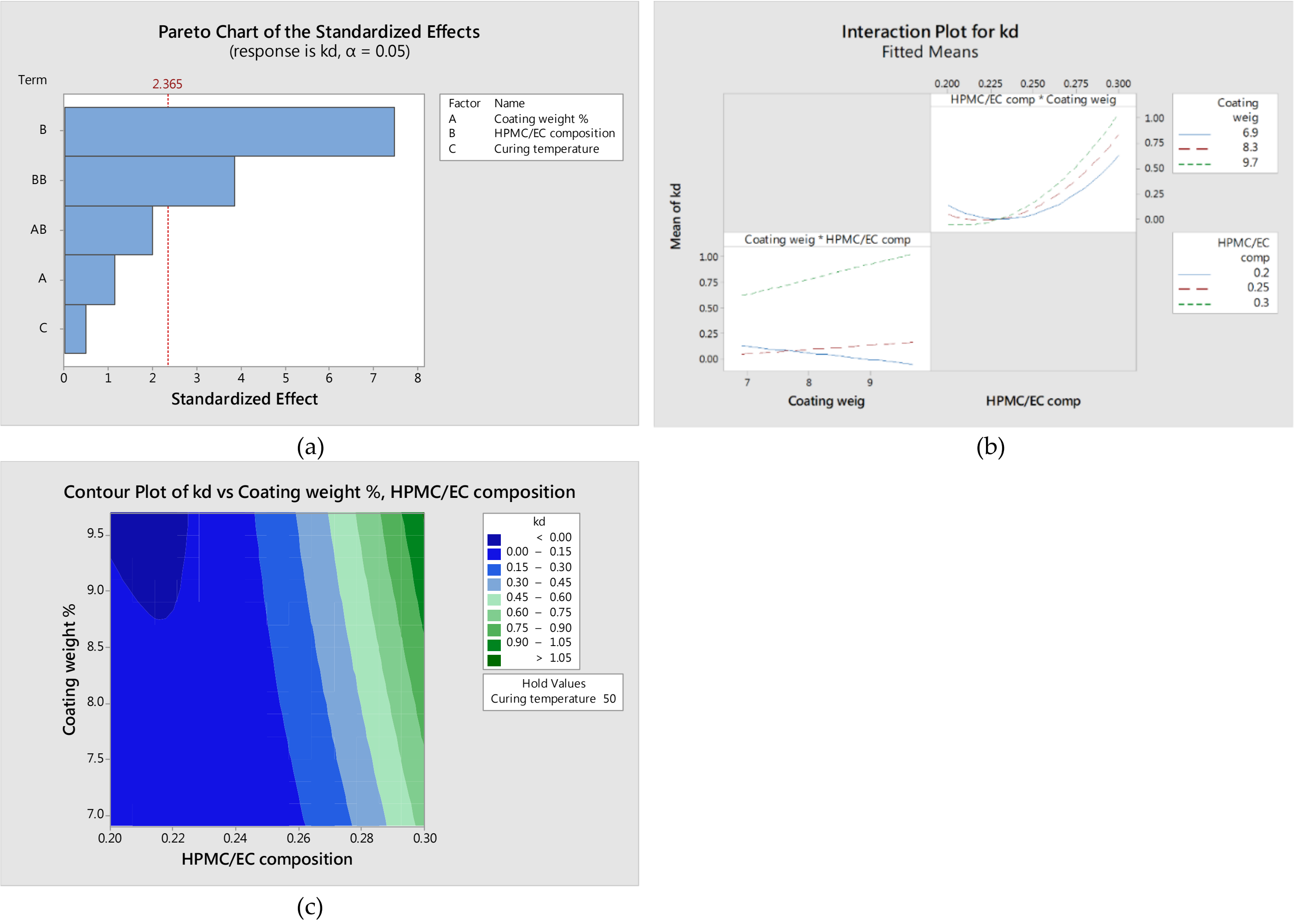

Figure S2. Results for DoE of SR pellets: (a) the strong and weak factors for formulation and the conditions of SR pellets; (b) the interaction plot for k_d; (c) the contour plot of k_d.

5. Study on the drug release kinetics of SR pellets

The results of model fitting are shown in Table S5 and Figure S3. The first-order model was suitable for explaining the drug release of SR pellets in the 0–8-h interval. The zero-order model and the Higuchi model were more suitable for describing the initial drug release (i.e., the first 60% of the drug release).

Table S5. Types of kinetic models fitted for *in vitro* drug release profiles of sobrerol SR pellets.

Pellet code	Zero-order			First-order			Higuchi			Korsmeyer–Peppas			
	k_0	$[R^2]_{60\%}$	$[R^2]_{8h}$	k_1	$[R^2]_{60\%}$	$[R^2]_{8h}$	k_H	$[R^2]_{60\%}$	$[R^2]_{8h}$	k_{KP}	n	$[R^2]_{60\%}$	$[R^2]_{8h}$
SR-5	66.100	0.995	0.466	1.082	0.994	0.996	63.608	0.974	0.730	66.100	1.206	0.978	0.399
SR-10	62.400	0.997	0.531	0.978	0.991	0.996	59.651	0.964	0.784	62.400	1.120	0.992	0.489
SR-1	57.600	1.000	0.555	0.858	0.980	0.992	54.223	0.936	0.574	57.600	0.951	0.998	0.574
SR-6	27.137	0.998	0.774	0.393	0.985	0.997	36.908	0.899	0.800	26.270	0.926	0.997	0.800
SR-13	9.157	0.990	0.958	0.131	0.999	0.999	23.311	0.942	0.973	10.407	1.032	0.988	0.952
SR-12	7.653	0.996	0.986	0.115	0.992	0.992	22.379	0.916	0.953	7.667	0.981	0.996	0.988
SR-7	7.761	0.996	0.980	0.115	0.989	0.994	22.551	0.899	0.951	6.405	0.953	0.996	0.984
SR-3	6.877	0.992	0.987	0.096	0.996	0.996	20.135	0.932	0.949	5.960	0.899	0.994	0.994
SR-8	4.330	0.998	0.998	0.059	0.991	0.991	15.080	0.917	0.917	3.937	0.956	0.998	0.998
SR-4	3.683	0.998	0.998	0.048	0.997	0.997	12.942	0.934	0.934	4.057	1.031	0.996	0.996
SR-2	2.974	0.997	0.997	0.036	0.999	0.999	10.470	0.937	0.937	3.170	1.011	0.998	0.996
SR-9	2.948	0.996	0.996	0.036	0.998	0.998	10.385	0.937	0.937	3.185	1.016	0.995	0.995
SR-11	2.476	0.997	0.997	0.029	0.994	0.994	8.583	0.908	0.908	2.243	0.965	0.996	0.996

k_0: the release constant in the zero-order model; k_1: the release constant in the first-order model; k_H: the release constant in the Higuchi model; k_{KP}: the release constant in the Korsmeyer–Peppas model.

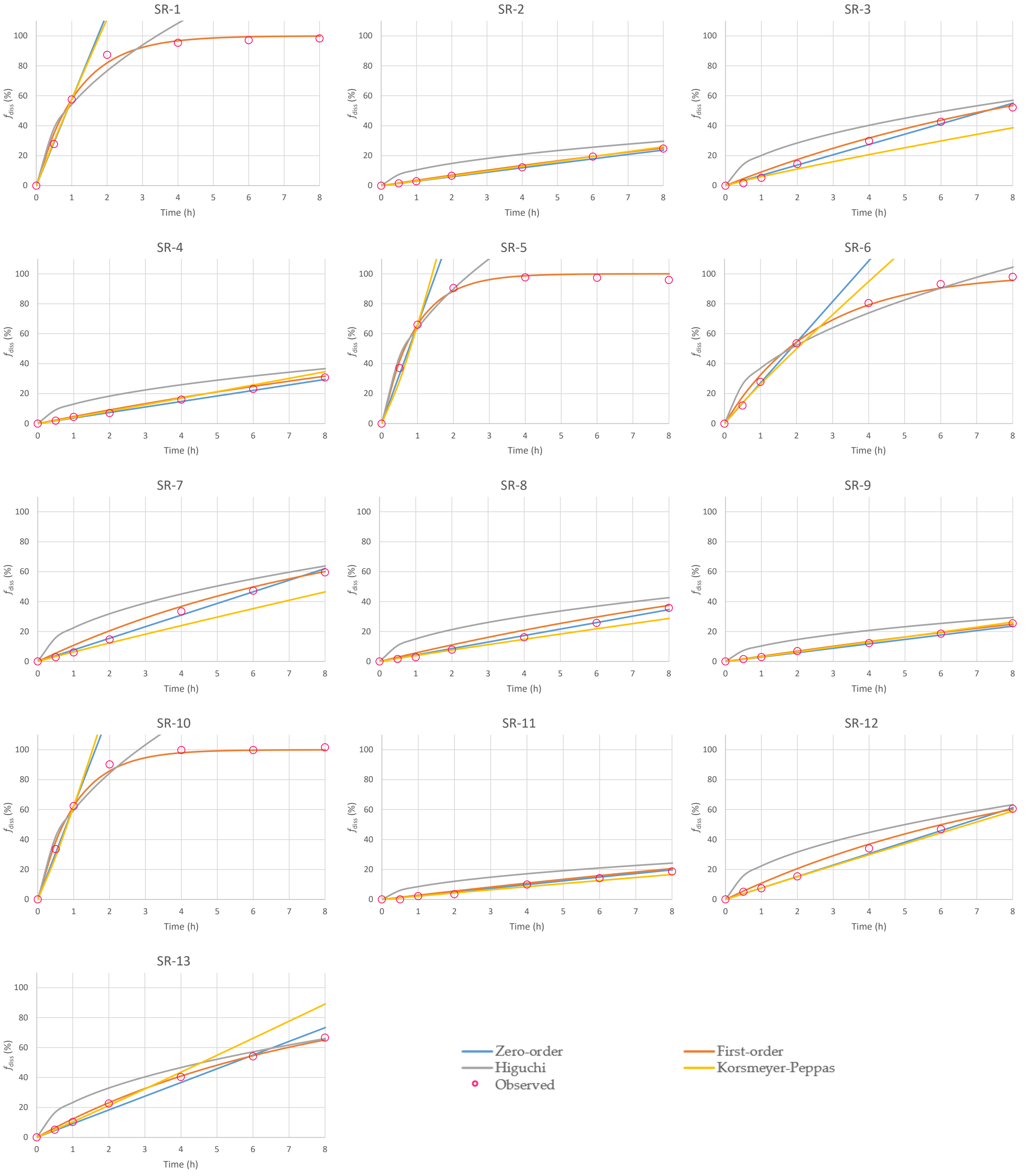

Figure S3. Fitting plots for 13 types of SR pellets with various kinetic models (i.e., zero-order model, first-order model, Higuchi model, and Korsmeyer–Peppas model).

6. Reconstruction of the sobrerol pharmacokinetic model with lag time

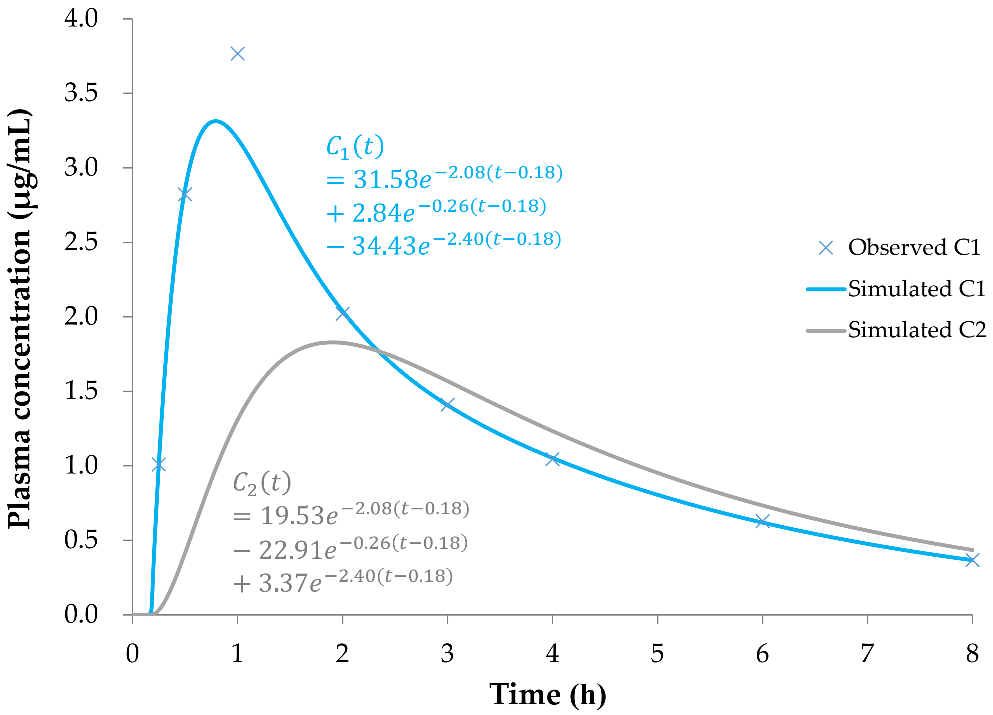

Figure S4. The oral two-compartment pharmacokinetic model of sobrerol with a t_{lag} of 0.18 h, which has more explanatory power in comparsion to the previous study in 1983.

7. Results for the second step of IVIVC

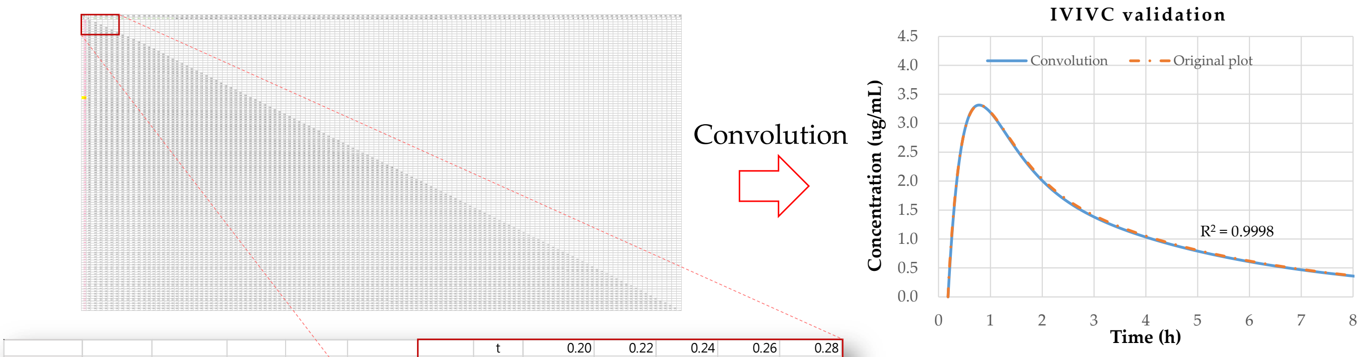

							t	0.20	0.22	0.24	0.26	0.28
t in-vitro (h)	t in-vivo (h)	fabs/fdiss (%)	D (ug)	ΔD	ΔC(ug/ml)	C1	ΔD	12295	13830	13165	12532	11930
0.00	0.18	0.00	0			0.00	0.18					
0.01	0.20	4.10	12295	12295	0.27	0.27	0.20	0.27				
0.02	0.22	8.71	26125	13830	0.31	0.56	0.22	0.27	0.31			
0.03	0.24	13.10	39291	13165	0.29	0.83	0.24	0.26	0.30	0.29		
0.04	0.26	17.27	51823	12532	0.28	1.09	0.26	0.25	0.29	0.28	0.28	
0.05	0.28	21.25	63753	11930	0.27	1.32	0.28	0.25	0.28	0.28	0.27	0.27

$$t_{\text{in-vitro}} = 0.494\, t_{\text{in-vivo}} - 0.0904$$

$$C_1(t) = \frac{D}{V_d}\left[\frac{(k_{21}-\alpha)}{(\beta-\alpha)}e^{-\alpha t} + \frac{(k_{21}-\beta)}{(\alpha-\beta)}e^{-\beta t}\right]$$

Figure S5. A reconverted pharmacokinetic profile obtained by convolution fitted well to the original one, showing that the IVIVC model of sobrerol was validated for use.

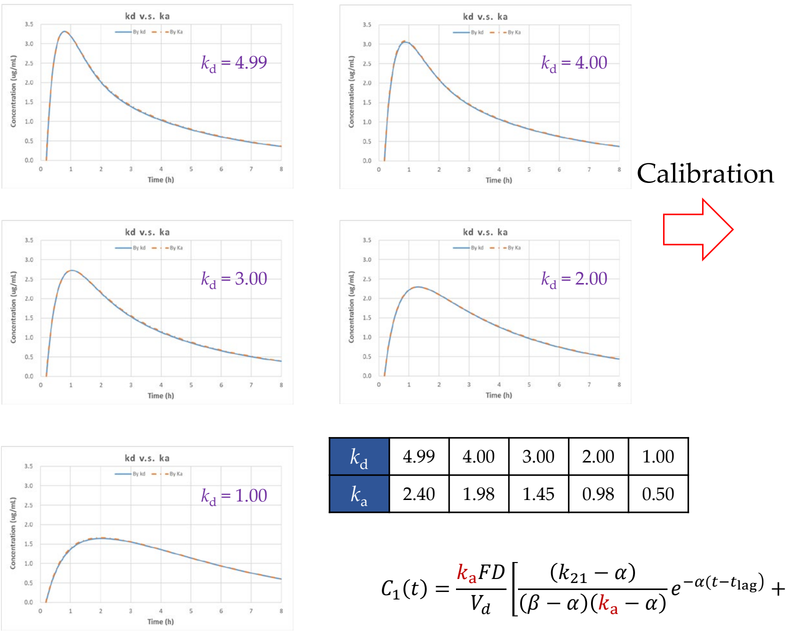

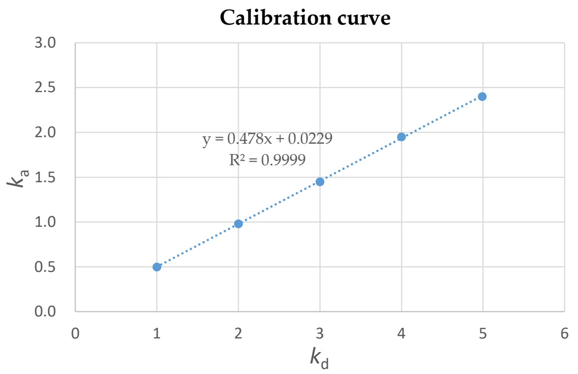

k_d	4.99	4.00	3.00	2.00	1.00
k_a	2.40	1.98	1.45	0.98	0.50

$$k_a = 0.478\, k_d + 0.0229$$

$$C_1(t) = \frac{k_a F D}{V_d}\left[\frac{(k_{21}-\alpha)}{(\beta-\alpha)(k_a-\alpha)}e^{-\alpha(t-t_{lag})} + \frac{(k_{21}-\beta)}{(k_a-\beta)(\alpha-\beta)}e^{-\beta(t-t_{lag})} + \frac{(k_{21}-k_a)}{(\alpha-k_a)(\beta-k_a)}e^{-k_a(t-t_{lag})}\right]$$

Figure S6. The calibration curve of k_d versus k_a was established using various simulated pharmacokinetic profiles convolved from sobrerol SR pellets with various rate constants of dissolution. Equation (1) can be further guided by k_d.
